# Population genomics reveals a candidate gene involved in bumble bee pigmentation

**DOI:** 10.1002/ece3.2935

**Published:** 2017-04-04

**Authors:** Meaghan L. Pimsler, Jason M. Jackson, Jeffrey D. Lozier

**Affiliations:** ^1^ Department of Biological SciencesUniversity of AlabamaTuscaloosaALUSA

**Keywords:** adaptation, *Bombus*, coloration, mimicry, RNAseq, SNPs

## Abstract

Variation in bumble bee color patterns is well‐documented within and between species. Identifying the genetic mechanisms underlying such variation may be useful in revealing evolutionary forces shaping rapid phenotypic diversification. The widespread North American species *Bombus bifarius* exhibits regional variation in abdominal color forms, ranging from red‐banded to black‐banded phenotypes and including geographically and phenotypically intermediate forms. Identifying genomic regions linked to this variation has been complicated by strong, near species level, genome‐wide differentiation between red‐ and black‐banded forms. Here, we instead focus on the closely related black‐banded and intermediate forms that both belong to the subspecies *B. bifarius nearcticus*. We analyze an RNA sequencing (RNAseq) data set and identify a cluster of single nucleotide polymorphisms (SNPs) within one gene, *Xanthine dehydrogenase/oxidase*‐like, that exhibit highly unusual differentiation compared to the rest of the sequenced genome. Homologs of this gene contribute to pigmentation in other insects, and results thus represent a strong candidate for investigating the genetic basis of pigment variation in *B. bifarius* and other bumble bee mimicry complexes.

## 1 Introduction

Pigment variation within and between populations and species has provided some of the most charismatic evidence for ecological and evolutionary forces involved in phenotypic differentiation (Kronforst et al., 2012). Although color variation can arise from neutral forces such as genetic drift, more often such patterns are the result of divergent selection on color morphs and can thus have implications for diverse processes from local adaptation to adaptive radiation of species (Rausher, 2008; Runemark, Hansson, Pafilis, Valakos, & Svensson, 2010; Wlasiuk, Carlos Garza, Lessa, & Smith, 2003). Insect pigmentation, in particular, has been studied in the context of adaptive strategies such as thermoregulation, crypsis, and warning coloration (Kronforst et al., 2012; True, 2003; Williams, 2007). Such color pattern variation is especially intriguing for its potential to reveal the genetic mechanisms of adaptive phenotypic change, and whether convergence across lineages occurs by shared versus independent molecular mechanisms (Oxford, 2005; Van Belleghem et al., 2017). For example, do species use the same basic tool sets to evolve pigmentation changes, co‐opt genes that serve related functions in other species in novel ways, or does convergent evolution occur through unique sets of genes in different lineages with similar phenotypes?

Bumble bees (Hymenoptera: Apidae: *Bombus*) commonly exhibit adaptive color pattern variation within and between species (Plowright & Owen, 1980; Williams, 2007). Co‐distributed species may converge on pigmentation patterns to produce Müllerian mimicry complexes (Müller, 1879), while intraspecific populations in different geographic regions can evolve different color patterns to match these local complexes (Hines & Williams, 2012; Plowright & Owen, 1980; Rapti, Duennes, & Cameron, 2014; Williams, 2007). Much of the flexibility appears to stem from partially independent “ground plan” color pattern elements that subdivide the abdomen inter‐ and intrasegmentally along anterioposterior and mediolateral axes, which are likely encoded by a combination of regulatory and downstream pigmentation genes (Rapti et al., 2014). Bumble bee color pattern complexes thus provide a model system in which to investigate genetic mechanisms underlying phenotypic polymorphism, the evolution and maintenance of aposematic coloration, and rapid phenotypic diversification.

Traditional genetic approaches for identifying molecular mechanisms underlying phenotypic variation is often challenging in bumble bees owing to difficulties of rearing and working with colonies in the laboratory (Plowright & Jay, 1966; Velthuis & van Doorn, 2006). Experimental research in bumble bees has thus often focused on a small set of species, especially domesticated species such as *B. impatiens* and *B. terrestris* (Amarasinghe, Clayton, & Mallon, 2014; Sadd et al., 2015; Stolle et al., 2011; Velthuis & van Doorn, 2006; Woodard et al., 2015) that may not be representative of the diversity of color pattern complexes in nature. Population genomics approaches have become a powerful tool for revealing adaptive genetic variation in nonmodel species where traditional genetics approaches are challenging and a priori knowledge of candidate genes is limited (Beaumont, 2005; Hohenlohe et al., 2010; Stapley et al., 2010). Phenotypically variable wild bumble bee populations are thus excellent targets for a population genomics approach (Lozier & Zayed, 2016), especially if discovered candidate genes can be cross‐validated against functional data from model organisms.

**Figure 1:**
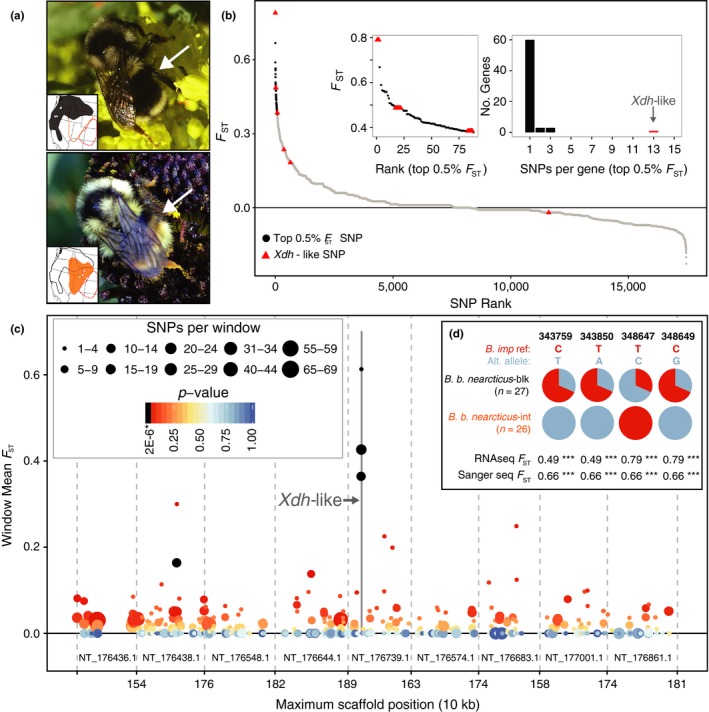
(a) Color patterns and distributions of *B. b. nearcticus*‐blk (top) and *nearcticus*‐int (bottom). RNAseq sample sites shown as white dots; *B. b. bifarius*‐red range indicated (red‐dashed line) for reference. (b) SNPs sorted by *F*_ST_, expanded view of 88 top‐0.5% SNPs (inset‐left), and histogram of top‐0.5% SNPs per gene (inset‐right), highlighting SNP's present in *Xdh‐*like (red triangles). (c) *F*_ST_‐per‐window across nine comparable scaffolds indicating SNP density and significance (see Section 2 and Appendix S2 for selection criteria). Three of five significant windows (see Fig. S4 for example test‐statistic distribution) overlap *Xdh‐like*; the comparison scaffold NT_176438.1 and has one (predicted triple functional domain protein); NT_176882.1 has the third (not shown) which encompasses three genes (predicted OTU domain‐containing protein 7B‐like, glutathione S‐transferase C‐terminal domain‐containing protein homolog, ubiquitin‐conjugating enzyme E2 R2). (d) Allele frequencies and *F*_ST_ (*** indicates *p *<* *.001) between *B. b. nearcticus*‐blk and *B. b. nearcticus*‐int at DNA sequencing‐validated SNP's

*Bombus* (*Pyrobombus*) *bifarius* Cresson is a widespread polymorphic species found most commonly at medium to high elevations throughout montane western North America (Stephen, 1957; Williams, Thorp, Richardson, & Colla, 2014). The species exhibits a striking red‐black dimorphism in abdominal coloration across its range (Lozier, Jackson, Dillon, & Strange, 2016; Stephen, 1957; Williams et al., 2014). Easternmost *B. bifarius* populations in the southern Colorado Rocky Mountains exhibit bright red pile on the second and third abdominal tergites (red‐banded subspecies *B. bifarius bifarius*‐red), while westernmost Pacific populations have black pile on these segments (black‐banded subspecies *B. bifarius nearcticus*‐blk). Geographically intermediate populations of *B. b. nearcticus* have a range of intermediate pigmentation, often exhibiting a clouded orange appearance from a variable mixture of pile colors (*B. b. nearcticus*‐int) (Lozier, Strange, & Koch, 2013; Lozier et al., 2016) (Figure 1a). The geographic distributions of these color patterns are consistent with a hypothesis of adaptive convergence on local color pattern complexes through Müllerian mimicry (Plowright & Owen, 1980; Thorp & Horning, 1983).

Divergent selection for red versus black abdominal pigmentation in *B. bifarius* might be expected to produce unique detectable differentiation patterns at genomic regions controlling color compared to the majority of the genome (i.e., “outlier” loci) (Beaumont, 2005; Stapley et al., 2010). Our previous analysis of relationships among red‐banded, black‐banded, and intermediate *B. bifarius* populations with genomewide data suggested unexpectedly large differentiation between *B. b. nearcticus* and *B. b. bifarius*‐red that is consistent with distinct species. Therefore, outlier loci were difficult to isolate from extensive neutral background divergence by comparing phenotypically extreme populations (i.e., *B. b. nearcticus*‐blk vs. *B. b. bifarius*‐red) (Lozier et al., 2016). However, weak genomewide differentiation among the much more closely related *B. b. nearcticus* populations may permit more sensitive tests to discover loci linked to phenotype, despite the less dramatic color differences (Figure 1a).

In this study, we reanalyze an existing transcriptome data set for *B. b. nearcticus* (Lozier et al., 2016) to identify outlier loci between black‐banded and intermediate color forms. We identify a cluster of SNPs exhibiting unusual behavior compared to the rest of the sequenced genome. Annotation with the sequenced *Bombus impatiens* genome (Sadd et al., 2015) reveals that SNPs all fall within *Xanthine dehydrogenase/oxidase*‐like (*Xdh‐*like), part of a gene family with established pigmentation roles in other animals (Frost, Borchert, Thorsteinsdottir, & Robinson, 1985; McGraw, 2005; Watt, 1972).

## 2 Methods

We utilize *B. bifarius* RNAseq data from Lozier et al. (2016) (doi:10.5061/dryad.k21r5; Appendix S1; Figure 1a) that includes samples of *B. b. nearcticus‐*blk (Oregon, USA), *B. b. nearcticus*‐int (Wyoming and Utah, USA), and *B. b. bifarius*‐red (Colorado, USA). We focus on RNAseq as a tool for characterizing SNPs from large numbers of gene regions (e.g., De Wit, Pespeni, & Palumbi, 2015). Briefly, RNA was extracted from the head and thorax of wild‐caught female bees preserved in RNAlater (Thermo Fisher Scientific Inc.; Waltham, MA USA) and sequenced with 100‐bp paired‐end reads on an Illumina HiSeq 2000 (Illumina, Inc.; San Diego, CA, USA). Reads were filtered and trimmed with TrimGalore (Krueger, 2015) to remove adaptor sequences, low‐quality bases (<20), and short reads (<20 bp). Trimmed reads were then mapped to the closely related *B. impatiens* (both subgenus *Pyrobombus*, common ancestry ~4–6 MYA; Hines, 2008) genome (Lozier et al., 2016; Sadd et al., 2015) using TopHat v2.0.10 (Kim et al., 2013). *Bombus impatiens* is the most closely related species to *B. bifarius* with a published genome (Cameron, Hines, & Williams, 2007), and *Bombus* as a whole exhibits a high degree of synteny between more distantly related species pairs (Sadd et al., 2015). Following suggested best practices (Quinn et al., 2013), reads with a map quality <20 were excluded and duplicate reads were removed with SAMTools (Li et al., 2009) and Picard (http://broadinstitute.github.io/picard/), respectively.

Variant calling was performed with SAMtools (mpileup, bcftools, and varFilter; Li et al., 2009), and indels and SNP's within 4 bp of a gap were excluded (Singhal (2013) see Lozier et al. (2016) for details). A final filtering with vcftools (Danecek et al., 2011) was performed for this study to include only those SNPs variable within *B. b. nearcticus* exons (annotated with snpEff v4.11: Cingolani et al., 2012), resulting in 17,450 SNPs for *B. b. nearcticus*‐blk (*N *=* *7) and *B. b. nearcticus*‐int (*N *=* *8). SNPs were identified in 261 *B. impatiens* scaffolds. Because whole chromosomes are not available in the *B. impatiens* assembly, for some comparisons and visual presentations, we examined the scaffold containing the discovered gene of interest (NT_176739.1) with respect to a subset of comparable scaffolds. We identified comparable scaffolds by first ranking scaffolds by length (±10 rank centered around NT_176739.1) and then by ranking this subset according to SNP density. We retained ±4 rank length‐selected scaffolds (ordered by ^Number of SNP's^/_Total length of scaffold_) around NT_176739.1 for visual presentation. Summary statistics for this scaffold set are provided in Appendix S2.

### 2.1 Population genetics and outlier analysis

The fixation index *F*_ST_ (Weir & Cockerham, 1984) and nucleotide diversity (π) were calculated per‐SNP and in sliding windows (10 kb windows, 5 kb increments) using vcftools v0.1.12b (Danecek et al., 2011). Tajima's *D* (Tajima, 1989) was calculated as an average across whole scaffolds and in nonoverlapping 10 kb windows that contained at least ten SNPs. Narrow windows are required because of rapid linkage disequilibrium decay in *Bombus* (Sadd et al., 2015). We tested for extreme window‐specific divergence using nested hierarchical bootstrapping to identify significantly elevated *F*_ST_ accounting for SNP density per window (similar to Hohenlohe et al., 2010), as follows. A null distribution of 500,000 pseudo‐replicate data sets was simulated. For each pseudo‐replicate, mean *F*_ST_ was calculated in windows matching the empirical data set by randomly sampling individual SNP *F*_ST_ values with replacement from the whole transcriptome data set, with the number of SNPs in the window corresponding to the number of SNPs observed in its corresponding empirical window. Simulations and figures used R version 3.2.4 (R Core team 2015; code is available on DRYAD). Given the large number of windows, we only considered a window as significant if no simulation produced a value as extreme as observed (*p *<* *2 × 10^−6^).

In addition to demonstrating that *Xdh*‐like SNPs are outliers within the genome using the sliding window *F*_ST_ bootstrap approach (Figure 1c), we also tested whether *Xdh*‐like SNPs were identified as outliers with a suite of several alternative methods that can identify targets of selection in wild populations while accounting for population structure. First, we used a clustering approach to identify SNPs contributing most to genetic divergence between *B. b. nearcticus‐*blk and *B. b. nearcticus*‐int using discriminant analysis of principal components (DAPC). The DAPC was performed for filtered synonymous/nonsynonymous RNAseq SNP set (*N *=* *17,450 loci) in the R package adegenet 2.0.1 (Jombart, 2008), grouping individuals into the two regional phenotypes, and retaining five principle components and a single discriminant axis. SNPs contributing most significantly to the discriminant analysis (“selected vs. unselected”) were identified by applying the snpzip function to the DAPC followed by hierarchical clustering using the “average” clustering method (hclust function).

A second principal components analysis method, pcadapt, is specifically designed to detect adaptive outliers in the face of population structure (Duforet‐Frebourg, Luu, Laval, Bazin, & Blum, 2016). The pcadapt R package uses principal components to control for population structure (in this case, we retained a single principal component for analysis based on initial evaluation with larger *K*, as above) and performs well under island models and models of divergence with admixture (high power, low false‐positive errors) (Duforet‐Frebourg et al., 2016). We implemented the analysis using default settings, excluding 8,314 SNPs with minor allele frequencies (MAF) < 0.05. To detect outliers, we employed a false discovery rate (FDR) correction with the R package qvalue (Storey, Bass, Dabney, & Robinson, 2016) with a FDR cutoff of 5%.

We next applied two outlier detection methods implemented in Arlequin 3.5 (Excoffier & Lischer, 2010), also filtered to remove loci with MAF < 0.05. The first approach is based on identifying outliers in a simulated distribution (100,000 coalescent simulations) of *F*_ST_ as a function of heterozygosity under the finite island model of 100 demes (FDIST approach in Appendix S3; similar to Beaumont & Balding, 2004). The second approach is similar, but relies on a hierarchical model of population structure—here using four groups (two times the number sampled) with 100 demes each—and has a reduced false‐positive rate under several more realistic demographic scenarios and should be more conservative (Excoffier, Hofer, & Foll, 2009) (HeirFDIST in Appendix S3). This approach has recently proven useful for detecting recent signatures of selection associated with recent divergence (e.g., Marques et al., 2016). For the hierarchical analysis, individuals were first grouped by site and then by regional color group. SNPs were removed from the hierarchical analysis if they were not present in any individual from a particular site.

Finally, we used a recently developed outlier detection method outFLANK (Whitlock & Lotterhos, 2015) that compares differentiation at each locus against a trimmed null distribution of *F*_ST_ values for loci not seemingly affected by strong selection. We removed loci with heterozygosity <0.1 and applied 5% trimming of the upper and lower *F*_ST_ distribution tails.

Because some results are presented spatially across scaffolds, to confirm structural conservation across our genome region of interest, we used Mauve (Darling, 2004) (implemented in Geneious R7; Kearse et al., 2012) to align NT_176739.1 from *B. impatiens* and orthologous scaffold NW_003566384.1 from *B. terrestris* (Sadd et al., 2015), assuming that strong synteny between these distantly related genomes (~20 mya) would reflect greater genome structure conservation between *B. bifarius* and *B. impatiens* (~4–6 mya) across the region of interest.

### 2.2 Sanger sequencing validation of SNPs

To confirm allele frequency differentiation at our target gene, including possible errors in the RNAseq data for this region from limited sampling, missing data, and incorrect SNP calling or read mapping, we performed Sanger DNA sequencing of four high‐*F*_ST_ *Xdh*‐like SNPs in a larger data set (53 individuals from 16 populations) (Appendix S1). Primer sets were designed using Geneious R7 such that one primer per pair sat within an intron and the other within an exon for the following loci: SNP's 348647 and 348649 with Xd_343538F (5′‐ TGTTGGAAGGCAGGTCAGTC‐3′**)** and Xd_343915R (5′‐AAGATAAGCCGTGCCACACA‐3′); SNP's 343850 and 343759 with Xd_348265F (5′‐CGCCAGTTACCAACCACTCT‐3′) and Xd_348680R (5′‐ACCGACCAATTCTGGATGCA‐3′). Amplification from specimens with available genomic DNA (modified DNeasy protocol, following Lozier, 2014; Qiagen, Valencia, CA) used the GoTaq^®^ G2 system (Promega, Madison, WI) with the following 25 μl reaction conditions [5 μl 5× PCR Buffer, 0.8–1 μl 25 mmol/L MgCl_2_, 0.5 μl 40 mmol/L (10 mmol/L each) dNTPs, 1 μl per primer (10 μmol/L each), 0.25 μl taq and 1–1.5 μl genomic DNA] and thermocycling conditions [5:00 denaturation at 95°C followed by 32–35 cycles of 95°C for 30 s, annealing at 52°C (for Xd_343538F/Xd_343915R) or 60–62°C (for Xd_348265F/Xd_348680R) for 30 s, and 72°C for 30 s, with a final 5‐min extension at 72°C]. PCR products were purified with ExoSAP‐IT (Affymetrix, Santa Clara, CA USA), and sequencing was performed at Eurofins Genomics (Louisville, KY USA). Sequences were manually inspected, edited, and aligned in Geneious R7 (Kearse et al., 2012). In all cases, heterozygotes were clearly identifiable from overlapping double peaks in the chromatograms. *F*_ST_ was calculated in Arlequin 3.5 (Excoffier & Lischer, 2010).

### 2.3 Phylogenetic analysis

We characterized phylogenetic relationships among *B. bifarius Xdh‐*like region sequences, including *B. b. bifarius*‐red, using statistical parsimony in the software TCS v1.21 (Clement, Posada, & Crandall, 2000). For this analysis, we extracted the RNAseq consensus sequences for the *Xdh‐*like region (NT_176739.1:340,370‐357,224) in IGV (Robinson et al., 2011), manually removed introns and other sites with missing data, and phased the sequences using the fastPHASE algorithm (Scheet & Stephens, 2006) in DNAsp v5.10.1 (Librado & Rozas, 2009), resulting in 41 complete sequences (40 *B. bifarius* haplotypes and the *B. impatiens* reference) of length 4,049 bp.

## 3 Results

Average differentiation for *B. b. nearcticus* is minimal (*F*_ST_ = 0.008 ± 0.078 *SD*), and few SNPs show a large degree of differentiation (*N* = 13, *F*_ST_ > 0.5 and *N* = 2, *F*_ST_ > 0.75) (Figure 1b). The two most highly differentiated SNPs map to *Xdh‐*like (LOC100741462 on NT_176739.1: 340,335–357,287 bp) (Appendices S1 and S3, Table S1). Of the top 0.5% highly differentiated SNPs (*N *=* *88, *F*_ST_ = 0.379–0.790), 13 (seven synonymous, six nonsynonymous) map to *Xdh‐*like, a notable outlier compared to other top‐*F*_ST_ genes (Figure 1b).

As expected from whole‐genome comparisons showing strong synteny in bumble bees (Sadd et al., 2015), NT_176739.1 structure is highly conserved between subgenera (Appendix S4), suggesting that SNP positioning from *B. impatiens* is suitable for *B. bifarius* (both subgenus *Pyrobombus*). A spike in differentiation in *Xdh‐*like is apparent for individual SNPs and windows (Figure 1c; Figs. S1–S4; Table S1). *F*_ST_ was significantly elevated (*p *<* *2 × 10^−6^) in all three windows overlapping *Xdh*‐like. *Xdh‐*like SNPs were recovered as significant in multiple outlier detection approaches (Appendix S3; Fig. S1), and no comparable patterns are observed elsewhere in the transcriptome. Tajima's *D* is also elevated in *Xdh*‐like (Fig. S3). *F*_ST_ from sequencing‐validated SNPs (*F*_ST_ = 0.66; Figure 1d, Table S2) were essentially identical to RNAseq values (average *F*_ST_ = 0.64), suggesting that RNAseq reliably estimates differentiation despite smaller sample sizes.

**Figure 2:**
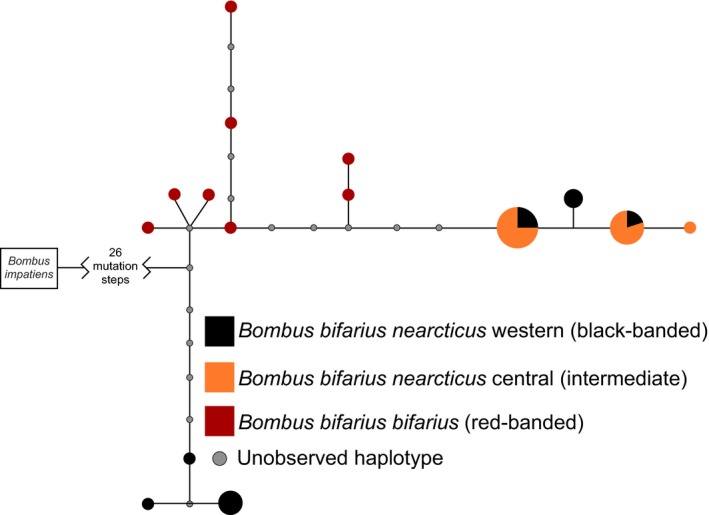
90% statistical parsimony TCS network for phased *Xdh*‐like sequences from the complete RNAseq data extracted for all *B. bifarius* subspecies (4,049 bp with no missing sequence). Colored circles represent unique haplotypes, shaded by population of origin (largest circle = 12 observed haplotypes, smallest = 1 haplotype) with unobserved mutation steps as small gray dots. More stringent haplotype networks produce the same topology within *B. bifarius*, and so 90% was selected to include the *B. impatiens* reference

Statistical parsimony analysis of 4,049 sites in the *Xdh*‐like region shows two groups of highly divergent *B. b. nearcticus* sequences, one set restricted to *B. b. nearcticus*‐blk, and another set including all *B. b. nearcticus*‐int and alleles from *B. b. nearcticus*‐blk heterozygotes. In contrast to previous work demonstrating that most of the genome shows *B. b. nearcticus* samples more closely related to each other than to *B. b. bifarius* (Lozier et al., 2016), both sets of these *Xdh*‐like sequences are approximately equally divergent from the *B. b. bifarius*‐red *Xdh‐*like sequences (Figure 2).

## 4 Discussion

Color pattern variation is among the most striking examples of adaptive phenotypic evolution that is excellently represented in the high degree of polymorphism within and between species of the genus *Bombus*. One of the major goals for investigating color variation in bumble bees is to understand the genetic mechanism underlying “color pattern elements” that facilitate adaptive divergence and convergence of color forms across the *Bombus* phylogeny (Rapti et al., 2014). Such loci have been identified in other color polymorphic mimicry complexes (e.g., *optix* in *Heliconius*) and are providing insights into evolutionary processes and genetic architectures underlying phenotypic variation (Kronforst et al., 2012; Van Belleghem et al., 2017). We show that *Xanthine dehydrogenase/oxidase‐*like contains unusual SNP density and divergence between *B. b. nearcticus* color variants. *Bombus b. nearcticus* is genetically well‐connected (Lozier et al., 2016), but *Xdh‐*like starkly contrasts with such genomewide patterns. *Xdh‐*like homologs are known to be involved in pigmentation (Frost et al., 1985; Watt, 1972; Yasukochi, Kanda, & Tamura, 1998). *Xdh*‐like is thus a strong candidate for one element contributing to bumble bee color pattern variation, representing a new example of selection using common pathways in novel evolutionary contexts.

*Xanthine dehydrogenase* and related genes code for molybdenum‐containing hydroxylases that catalyze purine and pteridine reactions (Brondino, Romão, Moura, & Moura, 2006). This family of genes contribute red, orange, and yellow pigmentation in many animals (Frost et al., 1985; Shamim, Ranjan, Pandey, & Ramani, 2014; Watt, 1972), including well‐known *Drosophila melanogaster* loci responsible for the classic red‐eyed phenotype (Reaume, Knecht, & Chovnick, 1991). In bumble bees, black, red, and yellow are the main pile colors (Rapti et al., 2014); black and red are melanins while yellow is likely a pterin (Hines, 2008). Results implicating a *Xanthine dehydrogenase* homolog thus suggest that the lighter reddish‐orange pigmentation in *B. b. nearcticus*‐int may be a unique state involving yellow, rather than a mix of pure red and black hairs. Although orange‐red tinged hairs are present in *B. b. nearcticus*‐int individuals, they are usually not as intense as in *B. b. bifarius*‐red bees, possibly indicating a mixture of pigments that includes pterins. This could also explain the lack of any noteworthy patterns across this genomic region from comparisons with *B. b. bifarius‐*red in our previous study (Lozier et al., 2016).

Comparisons across *B. bifarius* do provide some insights into the dynamics of phylogeographic divergence and phenotypic divergence within the species, although the importance of *Xdh*‐like in the evolution of the group remains to be fully understood. Bumble bee color pattern complexes are believed to be adaptive outcomes of Müllerian mimicry (Plowright & Owen, 1980; Williams, 2007). Color pattern may be more indicative of geography than phylogeny, as populations of the same species can diverge from one another and converge on a prominent local phenotype (Plowright & Owen, 1980). There is a clear relationship between geographic, phenotypic, and genetic divergence for *B. b. bifarius*‐red and *B. b. nearcticus*, although any signal at genes important for color pattern differentiation in this comparison is swamped by the extent of genomewide divergence (Lozier et al., 2016). In the case of the less extreme phenotypic differences within *B. b. nearcticus*, color variation is evident without differentiation across most of the genome, likely due to the greater range connectivity for these populations (Lozier et al., 2013). The broad geographic structure in coloration and in differentiation at *Xdh‐*like despite this demographic connectivity is consistent with strong selection that might be imposed by mimicry complexes. Nonetheless, there are regions of overlap for black‐banded and intermediate forms of *B. b. nearcticus*, especially in the north (e.g., Washington, Idaho), which, together with lack of fixation for *Xdh*‐like alleles in *B. b. nearcticus*‐blk (Figure 1d, SI), suggests that selection is imperfect. Parts of the *B. b. nearcticus* range overlap with other red/black color polymorphic species, for example, *B. melanopygous* (Plowright & Owen, 1980; Williams et al., 2014), and it may therefore be advantageous to maintain alleles associated with color pattern variation to facilitate convergence on local phenotypes (Williams, 2007). The unusual phylogenetic relationships among *Xdh*‐like haplotypes are particularly intriguing from this perspective (Figure 2), with the *B. b. nearcticus* haplotypes more divergent from each other than either is to *B. b. bifarius*‐red, potentially reflecting retention and modification of ancient alleles in the maintenance of phenotypic polymorphism. Alternatively, the partially overlapping distributions of phenotypes and *Xdh*‐like alleles in parts of the *B. b. nearcticus* range may simply reflect the balance between selection, migration, and drift. Additional sampling of finer scale transects, including sites where both *B. b. nearcticus* forms are present, should help refine these hypotheses.

One consideration is that *Xdh*‐like is a gene prediction, and its exact function in *B. bifarius* pigmentation thus remains uncertain. As discussed above, no SNPs perfectly segregate with color form; although intermediate *B. b. nearcticus*‐int populations were largely fixed for one allele at highly differentiated SNPs, this was not true in *B. b. nearcticus*‐blk. We were unable to confirm any obvious phenotypic associations between *B. b. nearcticus* genotype and abdominal or thoracic phenotype with post hoc examination of western *B. b. nearcticus* specimens with central *B. b. nearcticus* alleles. Sequenced SNPs may be incompletely linked to a causal structural or *cis‐*regulatory mutation that might show even stronger associations. However, causal or regulatory sites producing the unusual signatures at *Xdh‐*like detected here must lie near the sequenced SNPs due to weak linkage in bees (Sadd et al., 2015). The rapid decay of differentiation is highlighted by analysis of restriction site‐associated DNA sequences that show no elevated *F*_ST_ for intergenic SNPs flanking *Xdh*‐like (Fig. S2).

It is also unclear, at this point, how *Xdh*‐like alleles might interact with each other and other loci through dominance, epistasis, or regulatory differences. Additional work is thus necessary to determine which factors, either singly or in concert, are responsible for the development and maintenance of color pattern variation in *B. bifarius* and other bumble bee species. Examining differential expression of this gene and its alleles throughout development in different body segments will be a next step to determine how *Xdh*‐like contributes to color pattern variation (Mallarino et al., 2016). Experimental work with laboratory colonies will be necessary for functional genomics; however, maintaining successful laboratory colonies with bumble bees over multiple generations can be challenging (Plowright & Jay, 1966; Velthuis & van Doorn, 2006). Given the role of *Xdh‐*like homologs in pteridine production, screening for *Xdh*‐like outlier SNPs in other species complexes exhibiting color variation involving yellow pigmentation, such as *B. perplexus*,* B. terrestris*,* B. fervidus/B. californicus*, or *B. pensylvanicus*/*B. sonorous* (Williams et al., 2014), could provide complementary evidence for the gene's role in adaptive color pattern variation.

In conclusion, we have identified a gene that may facilitate understanding the formation of some color pattern complexes in bumble bees. Knowledge that *Xdh‐*like homologs affect pigmentation strengthens this hypothesis, but confirming mechanisms of action in *B. bifarius* will require additional experimentation. We also suspect that color in *B. bifarius* is likely to be complex, with multiple contributing loci. Our RNAseq data are likely to reveal only the strongest outlier loci, and by sequencing adult bees, will exclude sequence from genes expressed only during development. Developmental patterning genes (e.g., *Hox*‐genes) are likely key players in bumble bee color pattern evolution, for instance (Rapti et al., 2014). Finally, it will be important to consider ecological pressures driving color variation, and why selection produces certain complexes in particular geographic regions. Finer scale spatial sampling that incorporates whole‐genome data will help resolve some of these issues, while functional genomics and multispecies comparative approaches will be useful for targeting *Xdh*‐like's possible functions. Our findings provide a starting point for such studies of ontogenesis and evolution of color pattern in *B. bifarius* and other bumble bees.

## Data accessibility

Data, results, and analytical codes area available in the Supplementary Information, on DRYAD (http://dx.doi.org/10.5061/dryad.44c0r) and validation sequencing results are on NCBI Genbank (Nos. KX851406–KX851511).

## References

[ece32935-bib-0001] Amarasinghe, H. E. , Clayton, C. I. , & Mallon, E. B. (2014). Methylation and worker reproduction in the bumble‐bee (*Bombus terrestris*). Proceedings of the Royal Society of London, Series B: Biological Sciences, 281, 20132502. 24523266 10.1098/rspb.2013.2502 PMC4027386

[ece32935-bib-0002] Beaumont, M. (2005). Adaptation and speciation: What can *F* _ST_ tell us?. Trends in Ecology & Evolution, 20, 435–440. 16701414 10.1016/j.tree.2005.05.017

[ece32935-bib-0003] Beaumont, M. A. , & Balding, D. J. (2004). Identifying adaptive genetic divergence among populations from genome scans. Molecular Ecology, 13, 969–980. 15012769 10.1111/j.1365-294x.2004.02125.x

[ece32935-bib-0004] Brondino, C. D. , Romão, M. J. , Moura, I. , & Moura, J. J. (2006). Molybdenum and tungsten enzymes: The xanthine oxidase family. Current Opinion in Chemical Biology, 10, 109–114. 16480912 10.1016/j.cbpa.2006.01.034

[ece32935-bib-0005] Cameron, S. A. , Hines, H. M. , & Williams, P. H. (2007). A comprehensive phylogeny of the bumble bees (*Bombus*). Biological Journal of the Linnean Society, 91, 161–188.

[ece32935-bib-0006] Cingolani, P. , Platts, A. , Wang, L. L. , Coon, M. , Nguyen, T. , Wang, L. , … Ruden, D. M. (2012). A program for annotating and predicting the effects of single nucleotide polymorphisms, SnpEff. Fly, 6, 80–92. 22728672 10.4161/fly.19695 PMC3679285

[ece32935-bib-0007] Clement, M. , Posada, D. , & Crandall, K. A. (2000). TCS: A computer program to estimate gene genealogies. Molecular Ecology, 9, 1657–1659. 11050560 10.1046/j.1365-294x.2000.01020.x

[ece32935-bib-0008] Danecek, P. , Auton, A. , Abecasis, G. , Albers, C. A. , Banks, E. , DePristo, M. A. , Handsaker, R. E. , … McVean, G. (2011). The variant call format and VCF tools. Bioinformatics, 27, 2156–2158. 21653522 10.1093/bioinformatics/btr330 PMC3137218

[ece32935-bib-0009] Darling, A. C. E. (2004). Mauve: Multiple alignment of conserved genomic sequence with rearrangements. Genome Research, 14, 1394–1403. 15231754 10.1101/gr.2289704 PMC442156

[ece32935-bib-0010] De Wit, P. , Pespeni, M. H. , & Palumbi, S. R. (2015). SNP genotyping and population genomics from expressed sequences—Current advances and future possibilities. Molecular Ecology, 24, 2310–2323. 25808983 10.1111/mec.13165

[ece32935-bib-0011] Duforet‐Frebourg, N. , Luu, K. , Laval, G. , Bazin, E. , & Blum, M. G. B. (2016). Detecting genomic signatures of natural selection with principal component analysis: Application to the 1000 genomes data. Molecular Biology and Evolution, 33, 1082–1093. 26715629 10.1093/molbev/msv334 PMC4776707

[ece32935-bib-0012] Excoffier, L. , Hofer, T. , & Foll, M. (2009). Detecting loci under selection in a hierarchically structured population. Heredity, 103, 285–298. 19623208 10.1038/hdy.2009.74

[ece32935-bib-0013] Excoffier, L. , & Lischer, H. E. (2010). A new sereis of programs to perform population genetic analyses under Linux and Windows. Molecular Ecology Resources, 10, 564–567. 21565059 10.1111/j.1755-0998.2010.02847.x

[ece32935-bib-0014] Frost, S. K. , Borchert, M. E. , Thorsteinsdottir, S. , & Robinson, S. J. (1985). Xanthine dehydrogenase‐activity as a modulator of pigment cell‐differentiation. American Zoologist, 25, A92.

[ece32935-bib-0015] Hines, H. M. (2008). Bumble bees (Apidae: Bombus) through the ages: Historical biogeography and the evolution of color diversity. Urbana‐Champaign: University of Illinois.

[ece32935-bib-0016] Hines, H. M. , & Williams, P. H. (2012). Mimetic colour pattern evolution in the highly polymorphic *Bombus trifasciatus* (Hymenoptera: Apidae) species complex and its comimics: Evolution of mimicy in bumblebees. Zoological Journal of the Linnean Society, 166, 805–826.

[ece32935-bib-0017] Hohenlohe, P. A. , Bassham, S. , Etter, P. D. , Stiffler, N. , Johnson, E. A. , & Cresko, W. A. (2010). Population genomics of parallel adaptation in threespine stickleback using sequenced RAD tags. PLoS Genetics, 6, e1000862. 20195501 10.1371/journal.pgen.1000862 PMC2829049

[ece32935-bib-0018] Jombart, T. (2008). Adegenet: A R package for the multivariate analysis of genetic markers. Bioinformatics, 24, 1403–1405. 18397895 10.1093/bioinformatics/btn129

[ece32935-bib-0019] Kearse, M. , Moir, R. , Wilson, A. , Stones‐Havas, S. , Cheung, M. , Sturrock, S. , Buxton, S. , … Thierer, T. (2012). Geneious basic: An integrated and extendable desktop software platform for the organization and analysis of sequence data. Bioinformatics, 28, 1647–1649. 22543367 10.1093/bioinformatics/bts199 PMC3371832

[ece32935-bib-0020] Kim, D. , Pertea, G. , Trapnell, C. , Pimentel, H. , Kelley, R. , & Salzberg, S. L. (2013). TopHat2: Accurate alignment of transcriptomes in the presence of insertions, deletions and gene fusions. Genome Biology, 14, R36. 23618408 10.1186/gb-2013-14-4-r36 PMC4053844

[ece32935-bib-0021] Kronforst, M. R. , Barsh, G. S. , Kopp, A. , Mallet, J. , Monteiro, A. , Mullen, S. P. , … Hoekstra, H. E. (2012). Unraveling the thread of nature's tapestry: The genetics of diversity and convergence in animal pigmentation. Pigment Cell & Melanoma Research, 25, 411–433. 22578174 10.1111/j.1755-148X.2012.01014.x

[ece32935-bib-0022] Krueger, F. (2015). Trim Galore. A wrapper tool around Cutadapt and FastQC to consistently apply quality and adapter trimming to FastQ files. Retrieved from http://www.bioinformatics.babraham.ac.uk/projects/trim_galore/

[ece32935-bib-0023] Li, H. , Handsaker, B. , Wysoker, A. , Fennell, T. , Ruan, J. , Homer, N. , … Durbin, R. (2009). The sequence alignment/map format and SAMtools. Bioinformatics, 25, 2078–2079. 19505943 10.1093/bioinformatics/btp352 PMC2723002

[ece32935-bib-0024] Librado, P. , & Rozas, J. (2009). DnaSP v5: A software for comprehensive analysis of DNA polymorphism data. Bioinformatics, 25, 1451–1452. 19346325 10.1093/bioinformatics/btp187

[ece32935-bib-0025] Lozier, J. D. (2014). Revisiting comparisons of genetic diversity in stable and declining species: Assessing genome‐wide polymorphism in North American bumble bees using RAD sequencing. Molecular Ecology, 23, 788–801. 24351120 10.1111/mec.12636

[ece32935-bib-0026] Lozier, J. D. , Jackson, J. M. , Dillon, M. E. , & Strange, J. P. (2016). Population genomics of divergence among extreme and intermediate color forms in a polymorphic insect. Ecology and Evolution, 6, 1075–1091. 26811748 10.1002/ece3.1928 PMC4722823

[ece32935-bib-0027] Lozier, J. D. , Strange, J. P. , & Koch, J. B. (2013). Landscape heterogeneity predicts gene flow in a widespread polymorphic bumble bee, *Bombus bifarius* (Hymenoptera: Apidae). Conservation Genetics, 14, 1099–1110.

[ece32935-bib-0028] Lozier, J. D. , & Zayed, A. (2016). Bee conservation in the age of genomics. Conservation Genetics, 1–17, doi:10.1007/s10592‐016‐0893‐7

[ece32935-bib-0029] Mallarino, R. , Henegar, C. , Mirasierra, M. , Manceau, M. , Schradin, C. , Vallejo, M. , … Hoekstra, H. E. (2016). Developmental mechanisms of stripe patterns in rodents. Nature, doi:10.1038/nature20109 10.1038/nature20109 PMC5292240 27806375

[ece32935-bib-0030] Marques, D. A. , Lucek, K. , Meier, J. I. , Mwaiko, S. , Wagner, C. E. , Excoffier, L. , & Seehausen, O. (2016). Genomics of rapid incipient speciation in sympatric threespine stickleback. PLoS Genetics, 12, e1005887. 26925837 10.1371/journal.pgen.1005887 PMC4771382

[ece32935-bib-0031] McGraw, K. J. (2005). The antioxidant function of many animal pigments: Are there consistent health benefits of sexually selected colourants? Animal Behaviour, 69, 757–764.

[ece32935-bib-0032] Müller, F. (1879). *Ituna* and *Thyridia*: A remarkable case of mimicry in butterflies. Transactions of the Entomological Society of London, 1879, 20–29.

[ece32935-bib-0033] Oxford, G. S. (2005). Genetic drift within a protected polymorphism: Enigmatic variation in color‐morph frequencies in the candy‐stripe spider, *Enoplognatha ovata* . Evolution, 59, 2170–2184. 16405161 10.1554/05-046.1

[ece32935-bib-0034] Plowright, R. C. , & Jay, S. C. (1966). Rearing bumble bee colonies in captivity. Journal of Apicultural Research, 5, 155–165.

[ece32935-bib-0035] Plowright, R. C. , & Owen, R. E. (1980). The evolutionary significance of bumble bee color patterns: A mimetic interpretation. Evolution, 34, 622. 10.1111/j.1558-5646.1980.tb04002.x 28563986

[ece32935-bib-0036] Quinn, E. M. , Cormican, P. , Kenny, E. M. , Hill, M. , Anney, R. , Gill, M. , … Morris, D. W. (2013). Development of strategies for SNP detection in RNA‐seq data: Application to lymphoblastoid cell lines and evaluation using 1000 genomes data. PLoS One, 8, e58815. 23555596 10.1371/journal.pone.0058815 PMC3608647

[ece32935-bib-0037] R Core Team . (2015). R: A language and environment for statistical computing. R Foundation for Statistical Computing, Vienna, Austria. Retrieved from https://www.R-project.org/

[ece32935-bib-0038] Rapti, Z. , Duennes, M. A. , & Cameron, S. A. (2014). Defining the color pattern phenotype in bumble bees (*Bombus*): A framework for comparative understanding of development. Biological Journal of the Linnean Society, 113, 384–404.

[ece32935-bib-0039] Rausher, M. D. (2008). Evolutionary transitions in floral color. International Journal of Plant Sciences, 169, 7–21.

[ece32935-bib-0040] Reaume, A. G. , Knecht, D. A. , & Chovnick, A. (1991). The rosy locus in *Drosophila melanogaster*: Xanthine dehydrogenase and eye pigments. Genetics, 129, 1099–1109. 1783294 10.1093/genetics/129.4.1099 PMC1204774

[ece32935-bib-0041] Robinson, J. T. , Thorvaldsdottir, H. , Winckler, W. , Guttman, M. , Lander, E. S. , Getz, G. , & Mesirov, J. P. (2011). Integrative genomics viewer. Nature Biotechnology, 29, 24–26. 10.1038/nbt.1754 PMC3346182 21221095

[ece32935-bib-0042] Runemark, A. , Hansson, B. , Pafilis, P. , Valakos, E. D. , & Svensson, E. I. (2010). Island biology and morphological divergence of the Skyros wall lizard *Podarcis gaigeae*: A combined role for local selection and genetic drift on color morph frequency divergence? BMC Evolutionary Biology, 10, 269. 20813033 10.1186/1471-2148-10-269 PMC2939580

[ece32935-bib-0043] Sadd, B. B. M. , Barribeau, S. S. M. , Bloch, G. , de Graaf, D. C. , Dearden, P. , Elsik, C. G. , Gadau, J. , … Robertson, H. M. (2015). The genomes of two key bumblebee species with primitive eusocial organization. Genome Biology, 16, 1–32. 25908251 10.1186/s13059-015-0623-3 PMC4414376

[ece32935-bib-0044] Scheet, P. , & Stephens, M. (2006). A fast and flexible statistical model for large‐scale population genotype data: Applications to inferring missing genotypes and haplotypic phase. American Journal of Human Genetics, 78, 629–644. 16532393 10.1086/502802 PMC1424677

[ece32935-bib-0045] Shamim, G. , Ranjan, S. K. , Pandey, D. M. , & Ramani, R. (2014). Biochemistry and biosynthesis of insect pigments. European Journal of Entomology, 111, 149–164.

[ece32935-bib-0046] Singhal, S. (2013). De novo transcriptomic analyses for non‐model organisms: An evaluation of methods across a multi‐species data set. Molecular Ecology Resources, 13, 403–416. 23414390 10.1111/1755-0998.12077

[ece32935-bib-0047] Stapley, J. , Reger, J. , Feulner, P. G. D. , Smadja, C. , Galindo, J. , Ekblom, R. , … Slate, J. (2010). Adaptation genomics: The next generation. Trends in Ecology & Evolution, 25, 705–712. 20952088 10.1016/j.tree.2010.09.002

[ece32935-bib-0048] Stephen, W. P. (1957). Bumble Bees of Western America (Hymenoptera: Apoidea). Corvallis: Agricultural Experiment Station, Oregon State College.

[ece32935-bib-0049] Stolle, E. , Wilfert, L. , Schmid‐Hempel, R. , Schmid‐Hempel, P. , Kube, M. , Reinhardt, R. , & Moritz, R. F. A. (2011). A second generation genetic map of the bumblebee *Bombus terrestris* (Linnaeus, 1758) reveals slow genome and chromosome evolution in the Apidae. BMC Genomics, 12, 48. 21247459 10.1186/1471-2164-12-48 PMC3034698

[ece32935-bib-0050] Storey, J. D. , Bass, A. J. , Dabney, A. , & Robinson, D. (2016). qvalue: Q‐value estimation for false discovery rate control. R package version 3.2.4. Retrieved from http://github.com/jdstorey/qvalue

[ece32935-bib-0051] Tajima, F. (1989). Statistical method for testing the neutral mutation hypothesis by DNA polymorphism. Genetics, 123, 585–595. 2513255 10.1093/genetics/123.3.585 PMC1203831

[ece32935-bib-0052] Thorp, R. W. , Horning, D. S. & Dunning, L. L. (1983). Bumble bees and cuckoo bumble bees of California (Hymenoptera, Apidae). Bullentin of the California Insect Survey, v. 23. Berkeley, CA, USA: University of California Press.

[ece32935-bib-0053] True, J. R. (2003). Insect melanism: The molecules matter. Trends in Ecology & Evolution, 18, 640–647.

[ece32935-bib-0054] Van Belleghem, S. M. , Rastas, P. , Papanicolaou, A. , Martin, S. H. , Arias, C. F. , Supple, M. A. , … Hines, H. M. (2017). Complex modular architecture around a simple toolkit of wing pattern genes. Nature Ecology & Evolution, 1, 52. 10.1038/s41559-016-0052 PMC5432014 28523290

[ece32935-bib-0055] Velthuis, H. H. W. , & van Doorn, A. (2006). A century of advances in bumblebee domestication and the economic and environmental aspects of its commercialization for pollination. Apidologie, 37, 421–451.

[ece32935-bib-0056] Watt, W. B. (1972). Xanthine dehydrogenase and pteridine metabolism in *Colias* butterflies. Journal of Biological Chemistry, 247, 1445–1451. 4334999

[ece32935-bib-0057] Weir, B. S. , & Cockerham, C. C. (1984). Estimating F‐statistics for the analysis of population structure. Evolution, 38, 1358–1370. 10.1111/j.1558-5646.1984.tb05657.x 28563791

[ece32935-bib-0058] Whitlock, M. C. , & Lotterhos, K. E. (2015). Reliable detection of loci responsible for local adaptation: Inference of a null model through trimming the distribution of FST. American Naturalist, 186(Suppl), S24–S36. 10.1086/682949 26656214

[ece32935-bib-0059] Williams, P. H. (2007). The distribution of bumblebee colour patterns worldwide: Possible significance for thermoregulation, crypsis, and warning mimicry. Biological Journal of the Linnean Society, 92, 97–118.

[ece32935-bib-0060] Williams, P. H. , Thorp, R. W. , Richardson, L. L. , & Colla, S. R. (2014). Bumblebees of North America: An identification guide. Princeton, NJ, USA: Princeton University Press.

[ece32935-bib-0061] Wlasiuk, G. , Carlos Garza, J. , Lessa, E. P. , & Smith, T. B. (2003). Genetic and geographic differentiation in the Rio Negro tuco‐tuco (*Ctenomys rionegrensis*): Inferring the roles of migration and drift from multiple genetic markers. Evolution, 57, 913–926. 12778560 10.1111/j.0014-3820.2003.tb00302.x

[ece32935-bib-0062] Woodard, S. H. , Lozier, J. D. , Goulson, D. , Williams, P. H. , Strange, J. P. , & Jha, S. (2015). Molecular tools and bumble bees: Revealing hidden details of ecology and evolution in a model system. Molecular Ecology, 24, 2916–2936. 25865395 10.1111/mec.13198

[ece32935-bib-0063] Yasukochi, Y. , Kanda, T. , & Tamura, T. (1998). Cloning of two *Bombyx* homologues of the *Drosophila* rosy gene and their relationship to larval translucent skin colour mutants. Genetical Research, 71, 11–19. 9674379 10.1017/s0016672397003078

