# Supplementary material for: Population genomics reveals a candidate gene involved in bumble bee pigmentation
Source: Ecol Evol. 2017 Apr 4;7(10):3406–13. doi: 10.1002/ece3.2935 (PMC5433978; doi:10.1002/ece3.2935)
Supplement: Supplementary file 1 [file ECE3-7-3406-s001.docx]

Appendix 1. Collecting location site information for samples used in RNAseq (*N*(RNA)) and follow-up validation sequencing of genomic DNA (*N*(Val)).

| **Site** | ***N* (RNA)** | ***N***  **(Val)** | **Population** | **State** | **Latitude** | **Longitude** |
| --- | --- | --- | --- | --- | --- | --- |
| CA02.2014 |  | 4 | *nearcticus* west  (black-banded) | California | 41.361 | -122.200 |
| CA14.2015 |  | 4 | *nearcticus* west  (black-banded) | California | 36.596 | -118.736 |
| CA15.2015 |  | 5 | *nearcticus* west  (black-banded) | California | 37.202 | -119.214 |
| OR01.2012 | 2 | 2 | *nearcticus* west  (black-banded) | Oregon | 42.396 | -122.201 |
| OR02.2012 | 1 |  | *nearcticus* west  (black-banded) | Oregon | 45.301 | -121.769 |
| OR02.2014 |  | 2 | *nearcticus* west  (black-banded) | Oregon | 43.310 | -122.120 |
| OR03.2012 | 1 | 1 | *nearcticus* west  (black-banded) | Oregon | 45.331 | -121.708 |
| OR04.2012 | 2 | 2 | *nearcticus* west  (black-banded) | Oregon | 45.256 | -121.712 |
| OR04.2014 |  | 2 | *nearcticus* west  (black-banded) | Oregon | 45.312 | -121.648 |
| OR09.2012 | 1 | 1 | *nearcticus* west  (black-banded) | Oregon | 42.073 | -122.754 |
| OR10.2014 |  | 2 | *nearcticus* west  (black-banded) | Oregon | 42.076 | -122.717 |
| OR11.2014 |  | 2 | *nearcticus* west  (black-banded) | Oregon | 42.067 | -122.683 |
| UT01.2012 | 1 | 1 | *nearcticus* central (intermediate) | Utah | 41.735 | -111.823 |
| WY01.2012 |  | 3 | *nearcticus* central (intermediate) | Wyoming | 42.559 | -110.895 |
| WY02/03.2012 | 3 | 12 | *nearcticus* central (intermediate) | Wyoming | 43.657 | -110.797 |
| WY04.2012 | 3 | 9 | *nearcticus* central (intermediate) | Wyoming | 43.657 | -110.790 |
| WY06.2012 | 1 | 1 | *nearcticus* central (intermediate) | Wyoming | 43.669 | -110.821 |
