# Supplementary material for: Population genomics reveals a candidate gene involved in bumble bee pigmentation
Source: Ecol Evol. 2017 Apr 4;7(10):3406–13. doi: 10.1002/ece3.2935 (PMC5433978; doi:10.1002/ece3.2935)
Supplement: Supplementary file 2 [file ECE3-7-3406-s002.docx]

**Appendix 2**. Summary statistics of scaffolds selected for comparison to NT_176739.1 based on similarity in length and average SNP density in *B. bifarius nearcticus*.

| **Scaffold** | **Length (bp)** | **SNPs** | **SNP Density (^SNP^/_Kb_)** | **No. Genes** | **Mean *F*_ST_** | **Max *F*_ST_** | **Scaffold Tajima's D** | **Mean π per SNP**  **(All/West/Central)** |
| --- | --- | --- | --- | --- | --- | --- | --- | --- |
| NT_176436.1 | 1542087 | 263 | 0.17 | 52 | 0.0201 | 0.3607 | -1.11696 | 0.180/0.174/0.179 |
| NT_176438.1 | 1763384 | 256 | 0.15 | 150 | 0.0170 | 0.5618 | -1.36854 | 0.164/0.163/0.158 |
| NT_176548.1 | 1828789 | 208 | 0.11 | 106 | 0.0025 | 0.3854 | -1.36102 | 0.164/0.161/0.164 |
| NT_176644.1 | 1892912 | 209 | 0.11 | 145 | 0.0071 | 0.4228 | -1.1767 | 0.164/0.161/0.164 |
| NT_176739.1 | 1637695 | 167 | 0.10 | 160 | 0.0463 | 0.7902 | -1.21391 | 0.157/0.183/0.136 |
| NT_176574.1 | 1747843 | 163 | 0.09 | 141 | -0.0030 | 0.2394 | -1.29124 | 0.164/0.161/0.164 |
| NT_176683.1 | 1584682 | 124 | 0.08 | 92 | -0.0058 | 0.2693 | -0.999309 | 0.164/0.161/0.164 |
| NT_177001.1 | 1748612 | 131 | 0.07 | 147 | -0.0009 | 0.2571 | -1.1868 | 0.175/0.193/0.159 |
| NT_176861.1 | 1816176 | 125 | 0.07 | 57 | 0.0008 | 0.3388 | -1.18837 | 0.164/0.161/0.164 |
