# Supplementary material for: Population genomics reveals a candidate gene involved in bumble bee pigmentation
Source: Ecol Evol. 2017 Apr 4;7(10):3406–13. doi: 10.1002/ece3.2935 (PMC5433978; doi:10.1002/ece3.2935)
Supplement: Supplementary file 3 [file ECE3-7-3406-s003.docx]

Appendix 3. Comparison of outlier tests for the highly differentiated SNPs in the *Xanthine dehydrogenase/oxidase-*like gene. Columns from left to right: *Bombus impatiens* 2.0 assmbly scaffold (Scaffold), SNP position (Position), nucelotide in *B. impatiens* (reference) and derived alleles, whether SNP has a synonymous (SYN) or non-synonymous (NON) substitution effect, Weir and Cockerham’s *F*_ST_ value between black and intermediate morphs of *B. bifarius nearcticus* (W&C *F*_ST_) and whole-genome rank based on decreasing *F*_ST_ (rank), whether a SNP was identified as selected based on Discriminant Analysis of Principal Components (DAPC) analysis (yes) with rank in parentheses, FDIST analysis in Arlequin (Arlequin-FDIST), hierarchical FDIST analysis (Arlequin-HeirFDIST), the significance of differentiation from outFLANK with outlier rank out of the full data set, and presence (yes) as one of 42 outliers detected with a false positive detection rate *q-*value of 5% by pcadapt.

| **Scaffold** | **Position** | **Allele** | | **Effect** | **W&C *F*_ST_**  **(rank)** | **DAPC Selected SNP*^a^*** | **Arlequin-FDIST *F*_ST_ (*P-value*)** | **Arlequin-HeirFDIST**  ***F*_ST_ (*P-value*)*^b^*** | **outFLANK**  **Upper-tail**  ***P*-value (rank)** | **pcadapt**  ***q* = 0.05** |
| --- | --- | --- | --- | --- | --- | --- | --- | --- | --- | --- |
|  |  | **Reference** | **Derived** |  |  |  |  |  |  |  |
| NT_176739.1 | 348647 | T | C | SYN | 0.790 (1) | yes (9) | 0.785 (1E-7) | NA | 3.56E-5 (1) | yes |
| NT_176739.1 | 348649 | C | G | NON | 0.790 (1) | yes (9) | 0.785 (1E-7) | NA | 3.56E-5 (1) | yes |
| NT_176739.1 | 343759 | C | T | NON | 0.487 (17) | yes (27) | 0.503 (1.3E-5) | NA | 0.001 (13) | yes |
| NT_176739.1 | 343850 | T | A | SYN | 0.487 (17) | yes (27) | 0.503 (1.3E-5) | NA | 0.001 (13) | yes |
| NT_176739.1 | 344201 | G | A | SYN | 0.487 (17) | yes (27) | 0.503 (1.3E-5) | NA | 0.001 (13) | yes |
| NT_176739.1 | 344678 | C | T | SYN | 0.487 (17) | yes (27) | 0.503 (1.3E-5) | NA | 0.001 (13) | yes |
| NT_176739.1 | 344999 | T | C | NON | 0.487 (17) | yes (27) | 0.503 (1.3E-5) | NA | 0.001 (13) | yes |
| NT_176739.1 | 345677 | T | C | NON | 0.487 (17) | yes (27) | 0.503 (1.3E-5) | NA | 0.001 (13) | yes |
| NT_176739.1 | 341903 | T | A | NON | 0.385 (82) | yes (34) | 0.406 (1.2E-3) | 0.654 (1.7E-3) | 0.004 (66) | yes |
| NT_176739.1 | 342206 | A | G | SYN | 0.385 (82) | yes (34) | 0.406 (1.2E-3) | 0.654 (1.7E-3) | 0.004 (66) | yes |
| NT_176739.1 | 342821 | C | T | NON | 0.385 (82) | yes (34) | 0.406 (1.2E-3) | 0.654 (1.7E-3) | 0.004 (66) | yes |
| NT_176739.1 | 343524 | G | A | SYN | 0.385 (82) | yes (34) | 0.406 (1.2E-3) | 0.654 (1.7E-3) | 0.004 (66) | yes |
| NT_176739.1 | 349032 | A | G | SYN | 0.385 (82) | yes (34) | 0.406 (1.2E-3) | 0.654 (1.7E-3) | 0.004 (66) | yes |

*^a^* indicates presence in top 90 (~0.5%) SNPs identified as contributing to DAPC structure, with rank in parentheses (equivalent loading scores shown as tied). Note that DAPC also identified a 14^th^ SNP within *Xdh*-like as structurally important (position 344333; rank 87 of 90; *F*_ST_ = 0.236).

*^b^* NA indicates that at least one site with only 1 bee had missing data for a particular locus in the hierarchical test
