# Supplementary material for: Population genomics reveals a candidate gene involved in bumble bee pigmentation
Source: Ecol Evol. 2017 Apr 4;7(10):3406–13. doi: 10.1002/ece3.2935 (PMC5433978; doi:10.1002/ece3.2935)
Supplement: Supplementary file 4 [file ECE3-7-3406-s004.docx]

**

**Appendix 4**. Mauve analysis demonstrating the conservation of the genome region containing *Xdh*-like (marked with *) across distantly related bumble bees with sequenced genomes. As expected from genome-wide patterns suggesting high structural conservation in bumble bees, NT_176739.1 in the *B. impatiens* genome is highly conserved with respect to the *B. terrestris* genome, with no rearrangements and predicted gene/mRNA identity, order, and spacing essentially identical between the two species. This suggests that positioning information from *B. impatiens* SNPs should be reliable for the chromosomal region containing *Xdh*-like.
