# Supplementary material for: Population genomics reveals a candidate gene involved in bumble bee pigmentation
Source: Ecol Evol. 2017 Apr 4;7(10):3406–13. doi: 10.1002/ece3.2935 (PMC5433978; doi:10.1002/ece3.2935)
Supplement: Supplementary file 5 [file ECE3-7-3406-s005.docx]

*Supporting Information*

**Population genomics reveals a candidate gene involved in bumble bee pigmentation**

Meaghan L. Pimsler*^1^, Jason M. Jackson^1^, and Jeffrey D. Lozier^1^

^1^Department of Biological Sciences, University of Alabama, Box 870344, Tuscaloosa, AL, USA 35487

*To whom correspondence should be addressed: Email: [mlpimsler@gmail.com](mailto:mlpimsler@gmail.com)

**This document contains:**

Supplementary Methods and Results

Supplementary References

Supplementary Tables (in order of reference in main text)

Supplementary Figures (in order of reference in main text)

**Supplementary Methods and Results**

***Supplementary Outlier Detection***

All outlier detection approaches identified two populations, with SNP’s in Xdh-like the major discriminating factor (Fig. S1A). SNPs within Xdh-like clearly contributed more to the DAPC axis than surrounding markers on NT_176739.1 (Fig. S1B), similar to FST (Fig. 1C in main text and Fig. S2). Of the 90 SNPs were classified as “selected” (0.52% of the total data set), 14 SNPs (six predicted non-synonymous) within the Xdh-like locus are identified within this candidate set (15.6%) (ranks of 13 shown in Appendix 3; full list of selected SNPs available on DRYAD). Pcadapt provided very similar results; this program identified a total of 42 significant SNPs at a false discovery rate of 5%, 13 of which are in Xdh-like (Appendix 3). No other clusters of SNPs within a genomic region were similarly identified as outliers. Both analytical approaches with Arlequin demonstrated that Xdh-like SNPs exhibited significant excess differentiation with (we report FST for the hierarchical test as recommended in the ARLEQUIN manual; Appendix 3). Overall results of the OUTflank method were similar to those obtained from other methods. The top two outlier SNPs were from the Xdh-like gene (right-tail P = 3.56 x 10-5), with a total of the same 13 Xdh-like SNPs in the 88 top 0.5% most differentiated loci (all outliers with P < 0.005; Appendix 1). Although SNPs were highly significant outliers in terms of p-values, the small sample sizes may be preventing any loci in the data set from meeting the 5% false-discovery q-value threshold applied by OUTflank, unlike pcadapt above. Nonetheless, combined with the other analyses presented here and the near identical differentiation values from confirmation sequencing of top-FST SNPs in 53 samples, and knowledge of Xdh-like homologues affecting pigmentation in other organisms, the P-values from OUTflank provide further support for interesting phenomena at this putative candidate gene with respect to the remaining sequenced genome (Appendix 1). Note that plots of ARLEQUIN and OUTflank SNP statistics were generally similar to the individual SNP FST plot (Fig. S1) and results are available on DRYAD.

***Restriction Site Associated DNA tag analysis supports weak linkage disequilibrium near* Xdh*-like and confirms weak differentiation across NT_176739.1***

We also examined a double-digest restriction site associated DNA tag (ddRAD) (Peterson et al. 2012) data set from *B. bifarius* (Lozier et al. 2016) to screen for SNPs within or near *Xhd-*like. The ddRAD data were modified from (Lozier et al. 2016), available on DRYAD (doi: 10.5061/dryad.k21r5). The original data set consisted of loci generated *de novo* in STACKS (Catchen et al. 2011). We reanalyzed the same sequence data aligned to the *B*. *impatiens* reference genome using GSNAP (Wu and Nacu 2010; Wu et al. 2016), with maximum mismatches = 5 and excluding indels or multiply mapping reads (96% ± 1% SD mapping success across 18 *B. b. nearcitus-*W and 16 *B. b. nearcticus-int*). The alignments were sorted and converted to BAM format in Picard Tools (http://broadinstitute.github.io/picard) using the *SortSam* tool, then indexed using the *index* function in Samtools (Li et al. 2009). We then called SNPs using STACKS *refmap.pl* and used STACKS *populations* to identify loci, allowing 20% missing data per SNP across individuals (Catchen et al. 2011). *F*_ST_ per-SNP was calculated as in the main text.

SNP-specific *F*_ST_’s across NT_176739.1 for RNAseq and ddRAD data are shown in Fig. S2. The average differentiation between the *B. b. nearcticus* populations was similar to the RNAseq data set (main text) and very low (*F*_ST_ = 0.009). 208 ddRAD SNPs were identified across scaffold NT_176739.1. No SNPs fell within *Xdh-*like, although one SNP was identified approximately 2000bp from the gene (position 338,127), but failed to show any notable elevation in *F*_ST_. This is consistent with the weak levels of linkage disequilibrium and high recombination rate observed in bumble bees and further supports the unusual patterns within the *Xdh­*-like gene as unique.

**Supplementary References**

Catchen, J. M., A. Amores, P. Hohenlohe, W. Cresko, J. H. Postlethwait, and D.-J. De Koning. 2011. Stacks: Building and Genotyping Loci De Novo From Short-Read Sequences. G3. 1:171–182.

Li, H., B. Handsaker, A. Wysoker, T. Fennell, J. Ruan, N. Homer, G. Marth, G. Abecasis, and R. Durbin. 2009. The sequence alignment/map format and SAMtools. Bioinformatics 25:2078–2079.

Lozier, J. D., J. M. Jackson, M. E. Dillon, and J. P. Strange. 2016. Population genomics of divergence among extreme and intermediate color forms in a polymorphic insect. Ecol. Evol. 6:1075–1091.

Peterson, B. K., J. N. Weber, E. H. Kay, H. S. Fisher, and H. E. Hoekstra. 2012. Double digest RADseq: An inexpensive method for de novo SNP discovery and genotyping in model and non-model species. PLoS One. 7:e0037135.

Wu, T. D., and S. Nacu. 2010. Fast and SNP-tolerant detection of complex variants and splicing in short reads. Bioinformatics 26:873–81.

Wu, T. D., J. Reeder, M. Lawrence, G. Becker, and M. J. Brauer. 2016. GMAP and GSNAP for genomic sequence alignment: Enhancements to speed, accuracy, and functionality. Stat. Genomics Methods Protoc. 283–334.

Supplementary Tables and Figures

Table S1. Summary of 85 SNPs with the top 0.5% largest *F*_ST_ values between *B. b. nearcticus*-blk and *nearcticus*-int, including scaffold, position within scaffold, *F*_ST_ value, rank (by decreasing *F*_ST_), LOC number and gene name. Bold rows indicate SNPs within the *Xdh*-like candidate gene.

| Scaffold | Position | *F*_ST_ | Rank | LOC | Gene name |
| --- | --- | --- | --- | --- | --- |
| **NT_176739.1** | **348649** | **0.790181** | **1** | **LOC100741462** | **xanthine dehydrogenase/oxidase-like** |
| **NT_176739.1** | **348647** | **0.790181** | **2** | **LOC100741462** | **xanthine dehydrogenase/oxidase-like** |
| NT_176463.1 | 1784290 | 0.668318 | 3 | LOC100742367 | GPN-loop GTPase 2 |
| NT_176730.1 | 31894 | 0.589249 | 4 | LOC100748880 | translocon-associated protein subunit alpha |
| NT_176510.1 | 919667 | 0.565104 | 5 | LOC100741570 | poly(U)-specific endoribonuclease homologue |
| NT_176483.1 | 289904 | 0.562419 | 6 | LOC100742970 | uncharacterized |
| NT_176438.1 | 1046369 | 0.561788 | 7 | LOC100741245 | triple functional domain protein |
| NT_176654.1 | 1317103 | 0.557789 | 8 | LOC100743587 | uncharacterized |
| NT_176740.1 | 180941 | 0.557789 | 9 | LOC105680747 | cGMP-dependent protein kinase |
| NT_176781.1 | 221971 | 0.541667 | 10 | LOC100745377 | vitellogenin-like |
| NT_177000.1 | 615079 | 0.529622 | 11 | LOC100744259 | DET1 homologue |
| NT_176910.1 | 252240 | 0.50858 | 12 | LOC100748652 | J domain-containing protein |
| NT_176455.1 | 152673 | 0.505406 | 13 | LOC100744278 | BAG domain-containing protein Samui-like |
| NT_176656.1 | 149103 | 0.5 | 14 | LOC100747365 | reversion-inducing cysteine-rich protein with Kazal motifs |
| NT_176636.1 | 3245958 | 0.498927 | 15 | LOC100742055 | E3 ubiquitin-protein ligase HUWE1 |
| NT_177499.1 | 628668 | 0.496521 | 16 | LOC100748903 | dnaJ homologue subfamily B member 11 |
| **NT_176739.1** | **343759** | **0.486631** | **17** | **LOC100741462** | **xanthine dehydrogenase/oxidase-like** |
| **NT_176739.1** | **343850** | **0.486631** | **18** | **LOC100741462** | **xanthine dehydrogenase/oxidase-like** |
| **NT_176739.1** | **344999** | **0.486631** | **19** | **LOC100741462** | **xanthine dehydrogenase/oxidase-like** |
| **NT_176739.1** | **345677** | **0.486631** | **20** | **LOC100741462** | **xanthine dehydrogenase/oxidase-like** |
| **NT_176739.1** | **344201** | **0.486631** | **21** | **LOC100741462** | **xanthine dehydrogenase/oxidase-like** |
| **NT_176739.1** | **344678** | **0.486631** | **22** | **LOC100741462** | **xanthine dehydrogenase/oxidase-like** |
| NT_177040.1 | 52684 | 0.483607 | 23 | LOC100745343 | tropomyosin-1 |
| NT_176945.1 | 1081691 | 0.47619 | 24 | LOC100740991 | uncharacterized |
| NT_177864.1 | 696594 | 0.475 | 25 | LOC100743083 | phosphatidylinositol 3-kinase regulatory subunit gamma |
| NT_176508.1 | 526276 | 0.474702 | 26 | LOC100747753 | ubiquitin-conjugating enzyme E2 H |
| NT_177059.1 | 51991 | 0.474702 | 27 | LOC100741678 | choline/ethanolamine kinase |
| NT_177386.1 | 269973 | 0.459359 | 28 | LOC100743641 | titin |
| NT_177064.1 | 270975 | 0.459359 | 29 | LOC100744578 | muscle M-line assembly protein unc-89 |
| NT_176667.1 | 59582 | 0.457494 | 30 | LOC100748288 | sialin |
| NT_178085.1 | 633 | 0.45386 | 31 | LOC105681682 | ankyrin-2-like |
| NT_176570.1 | 1053983 | 0.451187 | 32 | LOC100743010 | F-box only protein 9 |
| NT_176922.1 | 135176 | 0.448496 | 33 | LOC100747253 | tumor necrosis factor alpha-induced protein 8-like protein |
| NT_176936.1 | 1089404 | 0.447547 | 34 | LOC100746386 | mediator of RNA polymerase II transcription subunit 16 |
| NT_176882.1 | 1981268 | 0.442729 | 35 | LOC100743350 | spondin-1 |
| NT_177333.1 | 145462 | 0.441407 | 36 | LOC105681420 | uncharacterized |
| NT_176882.1 | 259273 | 0.441407 | 37 | LOC100743148 | rab GDP dissociation inhibitor beta |
| NT_176922.1 | 14532 | 0.440434 | 38 | LOC100747733 | hemocyte protein-glutamine gamma-glutamyltransferase-like |
| NT_176731.1 | 137424 | 0.438758 | 39 | LOC100742948 | adenylyltransferase and sulfurtransferase MOCS3 |
| NT_176737.1 | 1969894 | 0.435975 | 40 | LOC100742595 | pre-mRNA-splicing factor 38A |
| NT_176967.1 | 1152239 | 0.42658 | 41 | LOC100744937 | gamma-glutamyltranspeptidase 1-like |
| NT_176644.1 | 1703789 | 0.422833 | 42 | LOC100744292 | PDZ and LIM domain protein Zasp |
| NT_176962.1 | 935160 | 0.422479 | 43 | LOC100748734 | kunitz-type serine protease inhibitor Bt-KTI-like |
| NT_176636.1 | 3247934 | 0.421687 | 44 | LOC100742055 | E3 ubiquitin-protein ligase HUWE1 |
| NT_177083.1 | 445935 | 0.421603 | 45 | LOC100749653 | diacylglycerol kinase theta |
| NT_176882.1 | 791428 | 0.420829 | 46 | LOC100746936 | OTU domain-containing protein 7B-like |
| NT_176882.1 | 791476 | 0.420829 | 47 | LOC100746936 | OTU domain-containing protein 7B-like |
| NT_176552.1 | 730047 | 0.420829 | 48 | LOC100743008 | carboxy-terminal domain RNA polymerase II polypeptide A small phosphatase 1 |
| NT_176796.1 | 1527054 | 0.417278 | 49 | LOC100742390 | titin-like |
| NT_176967.1 | 1311487 | 0.417278 | 50 | LOC100744331 | mitochondrial import inner membrane translocase subunit Tim21 |
| NT_176910.1 | 313569 | 0.417278 | 51 | LOC100749483 | waprin-Phi1-like |
| NT_177089.1 | 513171 | 0.415896 | 52 | LOC100743601 | spectrin beta chain |
| NT_176439.1 | 1592653 | 0.415896 | 53 | LOC100742849 | grpE protein homologue, mitochondrial-like |
| NT_176913.1 | 269489 | 0.411775 | 54 | LOC100744184 | uncharacterized |
| NT_176438.1 | 1044766 | 0.410875 | 55 | LOC100741245 | triple functional domain protein |
| NT_177059.1 | 141784 | 0.409909 | 56 | LOC100747256 | tctex1 domain-containing protein 2-like |
| NT_176882.1 | 144606 | 0.409909 | 57 | LOC100740590 | uncharacterized |
| NT_176736.1 | 78026 | 0.409847 | 58 | LOC100744770 | uncharacterized |
| NT_176561.1 | 927142 | 0.406061 | 59 | LOC100748921 | uncharacterized |
| NT_176438.1 | 1046235 | 0.406061 | 60 | LOC100741245 | triple functional domain protein |
| NT_177386.1 | 258693 | 0.405568 | 61 | LOC100743641 | titin |
| NT_177386.1 | 258774 | 0.405568 | 62 | LOC100743641 | titin |
| NT_176680.1 | 117754 | 0.4 | 63 | LOC100740896 | uncharacterized |
| NT_176882.1 | 796432 | 0.4 | 64 | LOC100747056 | glutathione S-transferase C-terminal domain-containing protein homologue |
| NT_176882.1 | 791972 | 0.399929 | 65 | LOC100746936 | OTU domain-containing protein 7B-like |
| NT_176913.1 | 270893 | 0.399166 | 66 | LOC100744184 | uncharacterized |
| NT_177064.1 | 282783 | 0.396416 | 67 | LOC100744578 | muscle M-line assembly protein unc-89 |
| NT_176442.1 | 432831 | 0.396209 | 68 | LOC100743409 | eukaryotic translation initiation factor 4 gamma 2 |
| NT_176897.1 | 668519 | 0.394561 | 69 | LOC100745383 | deoxynucleotidyltransferase terminal-interacting protein 2 |
| NT_176561.1 | 3075322 | 0.394123 | 70 | LOC100744724 | mitochondrial inner membrane protein OXA1L |
| NT_177089.1 | 62718 | 0.394123 | 71 | LOC100744859 | putative glycerol kinase 5 |
| NT_176882.1 | 2198080 | 0.392463 | 72 | LOC100743948 | putative oxidoreductase GLYR1 homologue |
| NT_179893.1 | 594033 | 0.392463 | 73 | LOC100745074 | 28S ribosomal protein S29, mitochondrial |
| NT_176515.1 | 1097844 | 0.390395 | 74 | LOC100741451 | coiled-coil domain-containing protein 93 |
| NT_176808.1 | 2172276 | 0.388889 | 75 | LOC100749801 | uncharacterized protein C17orf59 homologue |
| NT_176515.1 | 1095974 | 0.388144 | 76 | LOC100745483 | phosphorylase b kinase gamma catalytic chain, skeletal muscle/heart isoform |
| NT_176910.1 | 445984 | 0.386273 | 77 | LOC100740628 | proteasome-associated protein ECM29 homologue |
| NT_177386.1 | 65303 | 0.386273 | 78 | LOC105681462 | titin-like |
| NT_178085.1 | 28474 | 0.386273 | 79 | LOC100742486 | ankyrin-3-like |
| NT_176548.1 | 973417 | 0.385366 | 80 | LOC100744680 | protein turtle |
| NT_176936.1 | 1454445 | 0.385366 | 81 | LOC100744330 | testis-expressed sequence 2 protein |
| NT_177084.1 | 939042 | 0.384957 | 82 | LOC100747062 | alanine--glyoxylate aminotransferase 2-like |
| **NT_176739.1** | **342821** | **0.384957** | **83** | **LOC100741462** | **xanthine dehydrogenase/oxidase-like** |
| **NT_176739.1** | **349032** | **0.384957** | **84** | **LOC100741462** | **xanthine dehydrogenase/oxidase-like** |
| **NT_176739.1** | **343524** | **0.384957** | **85** | **LOC100741462** | **xanthine dehydrogenase/oxidase-like** |
| **NT_176739.1** | **341903** | **0.384957** | **86** | **LOC100741462** | **xanthine dehydrogenase/oxidase-like** |
| **NT_176739.1** | **342206** | **0.384957** | **87** | **LOC100741462** | **xanthine dehydrogenase/oxidase-like** |
| NT_178641.1 | 1600844 | 0.378479 | 88 | LOC100745678 | ATP-dependent RNA helicase DDX42 |

**Table S2.** Samples used for validation sequencing of highly divergent SNP’s in *Xanthine dehydrogenase-like* between color morphs of *Bombus bifarius nearcticus*. Columns from left to right: site ID (see Appendix 1 for site details), sample ID (bold if transcriptome was sequenced), population group and phenotype, and genotype of SNP’s 343759, 343850, 348647, and 348649 on scaffold NT_176739.1 (SNP Genotypes) using IUPAC ambiguity codes. Bold samples marked with * indicate transcriptomes that appear in Lozier *et al* (2016); genotypes are congruent between Sanger and RNAseq, where both DNA and RNA were available. *F*_ST_ for *B. b. nearcticus*-blk and *B. b. nearcticus*-int was the same for all SNPs = 0.66 (all *P* < 0.001), nearly identical to the average *F*_ST_ from the RNAseq SNPs (*F*_ST_ = 0.64) and equal or greater than all non-*Xdh*-like SNPs from the RNAseq data, suggesting the RNAseq data is producing reliable patterns of differentiation despite the smaller sample sizes (Fig. 1D). Variation for RNAseq across these SNPs appear to reflect patterns of missing RNAseq data for some bees, as in all samples where DNA and RNA isolations were available SNP allele states were identical between the data sets. In contrast to the weak linkage expected in bees, from the validation sequencing, *Xdh-*like seemingly exhibits high linkage across the resequenced SNPs: individuals were heterozygous across all four SNPs, or homozygous for all “black” or all “intermediate” populations alleles, with no evidence of recombinant haplotypes (also see Fig. 1D).

| **Site ID** | **Sample ID** | **Population** | **SNP Genotypes** |
| --- | --- | --- | --- |
| CA02.2014 | JDL694 | *nearcticus* west (black-banded) | TATG |
|  | JDL695 | *nearcticus* west (black-banded) | YWYS |
|  | JDL696 | *nearcticus* west (black-banded) | YWYS |
|  | JDL697 | *nearcticus* west (black-banded) | CTCC |
| CA14.2015 | JDL1230 | *nearcticus* west (black-banded) | CTCC |
|  | JDL1231 | *nearcticus* west (black-banded) | CTCC |
|  | JDL1232 | *nearcticus* west (black-banded) | CTCC |
|  | JDL1233 | *nearcticus* west (black-banded) | CTCC |
| CA15.2015 | JDL1257 | *nearcticus* west (black-banded) | YWYS |
|  | JDL1258 | *nearcticus* west (black-banded) | CTCC |
|  | JDL1259 | *nearcticus* west (black-banded) | CTCC |
|  | JDL1260 | *nearcticus* west (black-banded) | CTCC |
|  | JDL1261 | *nearcticus* west (black-banded) | CTCC |
| OR01.2012 | JDL125 | *nearcticus* west (black-banded) | YWYS |
|  | JDL126 | *nearcticus* west (black-banded) | TATG |
| OR02.2014 | JDL719 | *nearcticus* west (black-banded) | YWYS |
|  | JDL720 | *nearcticus* west (black-banded) | CTCC |
| OR03.2012 | **JDL133*** | *nearcticus* west (black-banded) | TATG |
| OR04.2012 | **JDL134*** | *nearcticus* west (black-banded) | TATG |
|  | JDL137 | *nearcticus* west (black-banded) | YWYS |
| OR04.2014 | JDL749 | *nearcticus* west (black-banded) | YWYS |
|  | JDL750 | *nearcticus* west (black-banded) | YWYS |
| OR09.2012 | **JDL211*** | *nearcticus* west (black-banded) | CTCC |

**Table S2 continued**

| **Site ID** | **Sample ID** | **Population** | **SNP Genotypes** |
| --- | --- | --- | --- |
| OR10.2014 | JDL867 | nearcticus west (black-banded) | CTCC |
|  | JDL868 | nearcticus west (black-banded) | YWYS |
| OR11.2014 | JDL912 | nearcticus west (black-banded) | YWYS |
|  | JDL913 | nearcticus west (black-banded) | CTCC |
| UT01.2012 | **JDL414*** | nearcticus central (intermediate) | TATG |
| WY01.2012 | JDL348 | nearcticus central (intermediate) | TATG |
|  | JDL350 | nearcticus central (intermediate) | TATG |
|  | JDL351 | nearcticus central (intermediate) | TATG |
| WY02.2012 | JDL353 | nearcticus central (intermediate) | TATG |
|  | JDL352 | nearcticus central (intermediate) | TATG |
|  | JDL354 | nearcticus central (intermediate) | TATG |
| WY03.2012 | JDL356 | nearcticus central (intermediate) | TATG |
|  | JDL357 | nearcticus central (intermediate) | TATG |
|  | JDL365 | nearcticus central (intermediate) | TATG |
|  | JDL367 | nearcticus central (intermediate) | TATG |
|  | JDL369 | nearcticus central (intermediate) | TATG |
|  | JDL370 | nearcticus central (intermediate) | TATG |
|  | JDL372 | nearcticus central (intermediate) | TATG |
|  | JDL373 | nearcticus central (intermediate) | TATG |
|  | **JDL376*** | nearcticus central (intermediate) | TATG |
| WY04.2012 | JDL387 | nearcticus central (intermediate) | TATG |
|  | **JDL362*** | nearcticus central (intermediate) | TATG |
|  | JDL363 | nearcticus central (intermediate) | TATG |
|  | JDL384 | nearcticus central (intermediate) | TATG |
|  | **JDL385*** | nearcticus central (intermediate) | TATG |
|  | JDL386 | nearcticus central (intermediate) | TATG |
|  | JDL360 | nearcticus central (intermediate) | TATG |
|  | JDL361 | nearcticus central (intermediate) | TATG |
|  | JDL381 | nearcticus central (intermediate) | TATG |
| WY06.2012 | **JDL388*** | nearcticus central (intermediate) | TATG |


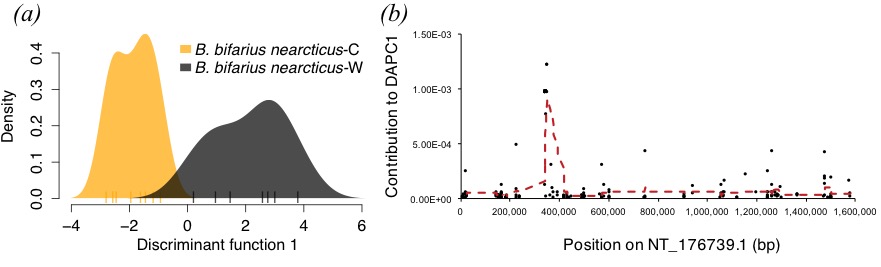


Figure S1. Example result from one alternative outlier detection method. (A) Density plot from discriminant analysis of principle components among *B. bifarius nearcticus-*blk and *B. bifarius nearcticus*-int individuals for 17,450 RNAseq-derived synonymous and non-synonymous SNPs (positions of individual bees along Discriminant function 1 shown as vertical hashes). (B) Contribution of each SNP along *B. impatiens* scaffold NT_176739.1 to the discriminant analysis of principle components among *B. bifarius nearcticus*-blk and *B. bifarius nearcticus*-int individuals for 17,450 RNAseq-derived synonymous and non-synonymous SNPs. A 10-SNP moving average line (red) highlights the unusual peak at the *Xdh-*like locus.

**Figure S2.** Plots of per-SNP *F*_ST_ for RNAseq derived (top) and ddRAD sequencing derived (bottom) SNPs for *B. bifarius nearcticus*-blk vs. *B. bifarius nearcticus*-int across *B. impatiens* scaffold NT_176739.1, highlighting the placement of *Xdh*-like in gray. Outside of *Xdh*-like patterns of differentiation are highly similar for the data sets. No SNPs were located within *Xdh*-like in the ddRAD data, however SNPs closely adjacent to the candidate gene show no unusual levels of differentiation, highlighting the narrow haplotypes in bumble bees and the uniqueness of the RNAseq *Xdh*-like region.


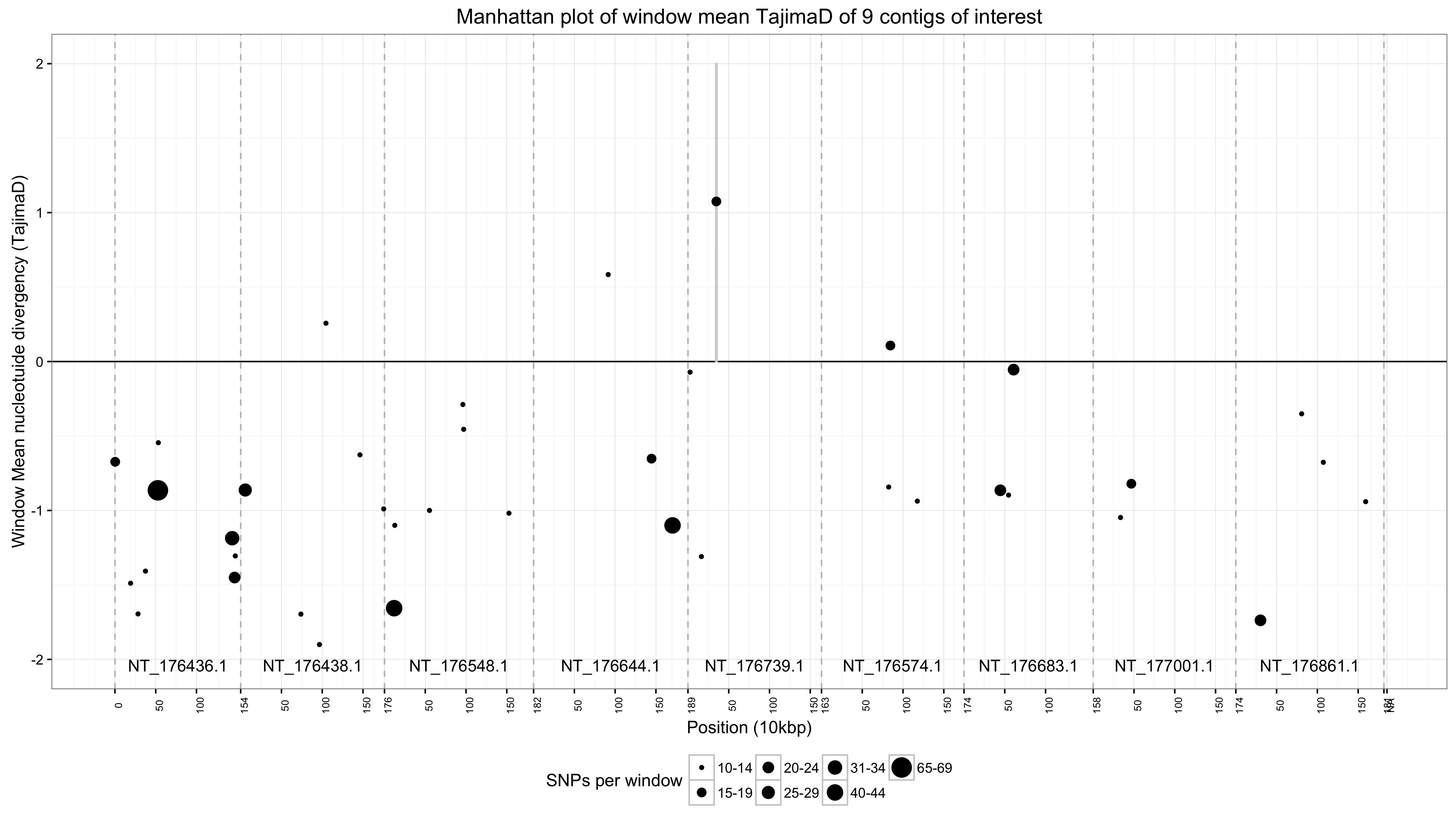


Figure S3. Plot of window mean Tajima’s D for 10 kb windows with at least 10 SNPs for all color morphs of *B. bifarius nearcticus* based on RNAseq SNPs. Position on scaffolds plotted on the x-axis, with window mean Tajima’s D plotted on the y-axis. The diameter of the dot indicates the number of SNPs in the window and the position of *xanthine dehydrogenase-like* is shown with a grey bar. Tajima’s D (Tajima 1989) was calculated for all samples pooled as a single population for 10Kb windows that contained at least 10 SNPs. The single *Xdh*-like overlapping window displays the largest Tajima’s D value across examined scaffolds. In the analysis of bees as a single pooled sample, an unusually large Tajima’s D likely suggests an excess of high-frequency variation in the region, mimicking a signature of balancing selection due to the fixation or near fixation of one set of alleles in *B. b. nearcticus*-int bees and a high frequency of the alternative alleles in *B. b. nearcticu*s-blk.


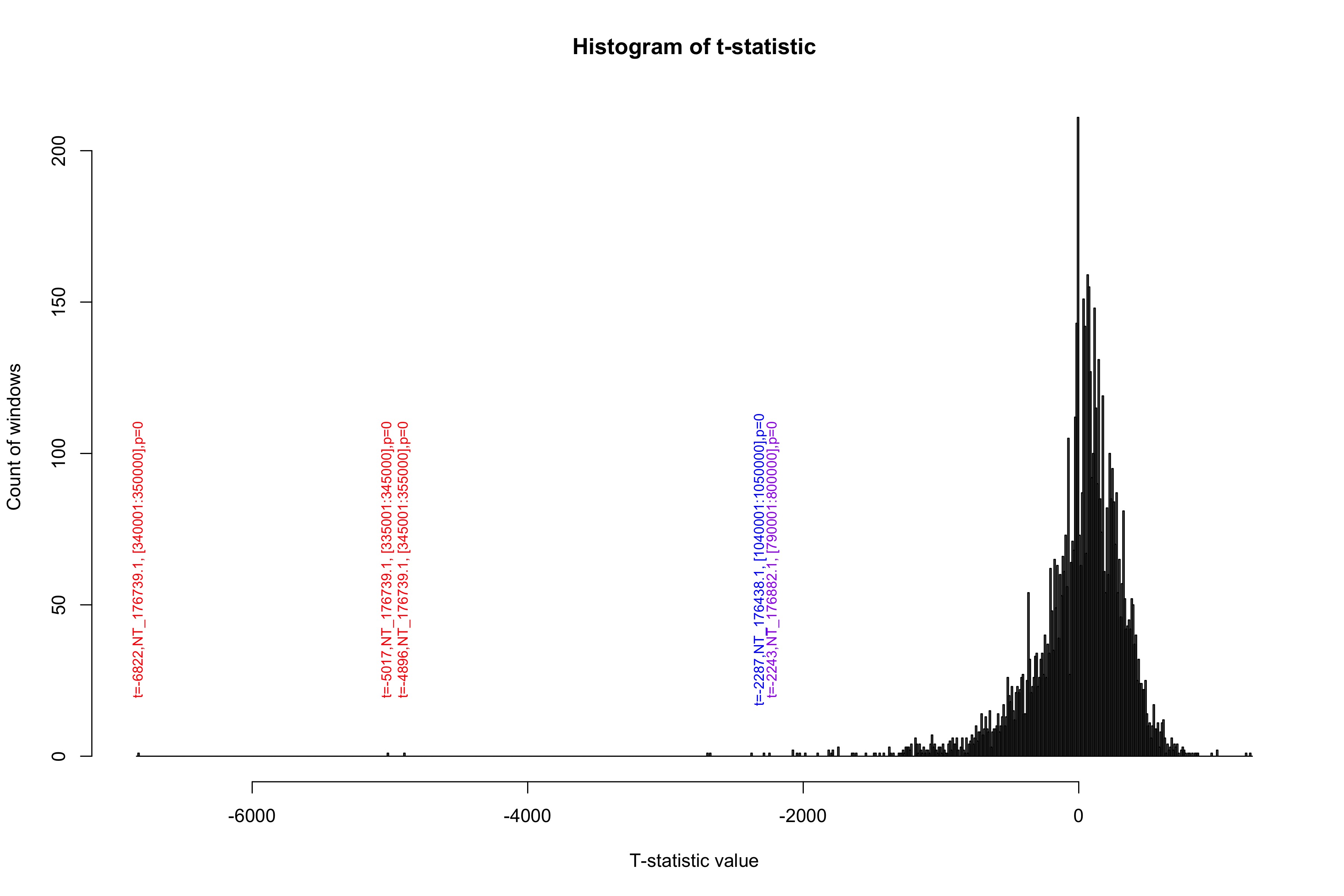


**Histogram of Test Statistic Values of Hierarchical Bootstrap Analysis**

Figure S4. Histogram of t-statistic values of hierarchically bootstrapped mean window *F*_ST_ values versus the observed mean *F*_ST_. The five windows with an empirical *P*-value of 0 for all 500,000 bootstraps are annotated on the graph with a different color for each scaffold. The three most extreme (red) windows are all from scaffold NT_176739.1 and all overlap *Xdh*-like*.*
